# Improvement of resistance to rice blast and bacterial leaf streak by CRISPR/Cas9-mediated mutagenesis of *Pi21* and *OsSULTR3;6* in rice (*Oryza sativa* L.)

**DOI:** 10.3389/fpls.2023.1209384

**Published:** 2023-07-17

**Authors:** Jinlian Yang, Yaoyu Fang, Hu Wu, Neng Zhao, Xinying Guo, Enerand Mackon, Haowen Peng, Sheng Huang, Yongqiang He, Baoxiang Qin, Yaoguang Liu, Fang Liu, Shengwu Chen, Rongbai Li

**Affiliations:** ^1^ State Key Laboratory for Conservation and Utilization of Subtropical Agro-Bioresources, College of Agriculture, Guangxi University, Nanning, China; ^2^ State Key Laboratory for Conservation and Utilization of Subtropical Agro-bioresources, College of Life Science and Technology, Guangxi University, Nanning, China; ^3^ State Key Laboratory for Conservation and Utilization of Subtropical Agricultural Bioresources, South China Agricultural University, Guangzhou, China; ^4^ Guangxi Lvhai Seed Co., Ltd, Marketing Department, Nanning, China

**Keywords:** rice, CRISPR/Cas9, *Pi21*, *OsSULTR3;6*, rice blast, bacterial leaf streak

## Abstract

Rice (*Oryza sativa* L.) is a staple food in many countries around the world, particularly in China. The production of rice is seriously affected by the bacterial leaf streak and rice blast, which can reduce rice yield or even cause it to fail to be harvested. In this study, susceptible material 58B was edited by CRISPR/Cas9, targeting a target of the *Pi21* gene and a target of the effector-binding element (EBE) of the *OsSULTR3;6* gene, and the mutants *58b* were obtained by Agrobacterium-mediated method. The editing efficiency of the two targets in the T_0_ generation was higher than 90.09%, the homozygous mutants were successfully selected in the T_0_ generation, and the homozygous mutation rate of each target was higher than 26.67%. The expression of the edited *pi21* and EBE of *Ossultr3;6* was significantly reduced, and the expression of defense responsive genes was significantly upregulated after infected with rice blast. The lesion areas of rice blast and bacterial leaf streak were significantly reduced in *58b*, and the resistance of both was effectively improved. Furthermore, the gene editing events did not affect the agronomic traits of rice. In this study, the resistance of *58b* to rice blast and bacterial leaf streak was improved simultaneously. This study provides a reference for using Clustered Regularly Interspaced Short Palindromic Repeats/Cas9 (CRISPR/Cas9) to accelerate the improvement of rice varieties and the development of new materials for rice breeding.

## Introduction

Rice (*Oryza sativa* L.) is one of the most important food crops in the world. Bacterial leaf streak and rice blast are two deadly diseases that can cause serious damage to rice (Ke et al., 2017). Bacterial leaf streak is a bacterial disease caused by *Xanthomonas oryzae pv.oryzicola* (*Xoc*) that mainly infects rice leaves through leaf stomata or wounds (Jiang et al., 2020). The genome similarity between *Xoc* and another *Xanthomonas oryzae* pv. *oryzae* (*Xoo*) is more than 90%, and they both lead to rice disease by introducing transcription activator–like effectors (TALEs) into plant cells to activate the expression of the susceptibility gene(Cox et al., 2017). TALE is a class of proteins unique to *Xanthomonas* species (Jens and Ulla, 2010; Boch et al., 2014). TALE can activate either the susceptibility gene (S) or the resistance gene (R) of the plant, thereby making the host susceptible or activating the defense mechanism of the pathogen (Boch et al., 2014). TALE contains a conserved central repeat region consisting of 34 amino acid repeats, an N-terminal region of the type III secretion system, and a C-terminal region containing transcriptional activation domains and nucleoplasm localization signals (Xu et al., 2022). So far, the targeting of TALE has been determined by the central repeat region, where each repeat unit recognizes a nucleotide through a specific degenerate codon, resulting in a contiguous DNA sequence [effector-binding element (EBE)] (Matthew and Adam, 2009; Jens and Ulla, 2010). *Magnaporthe oryzae*–caused rice blast is one of the most damaging diseases to rice, with a large damage area and severity (Liu et al., 2014). Rice blast can infect the leaves, stems, panicles, and roots of rice at various developmental stages (Wilson and Talbot, 2009), resulting in a significant decrease in rice yield (Ebbole, 2007). Following pathogen infection, rice plants activate the biosynthesis and signal transduction of various hormones that act as immune signals to activate host defense responses against pathogen invasion (David et al., 2013; Yang et al., 2013). Jasmonic acid (JA) enhances resistance to rice blast by activating defense-related genes and accumulating antimicrobial substances (Shiduku et al., 2014). It has been reported that the ubiquitin-proteasome system negatively regulates *OsWRKY45* through periodic degradation in the absence of pathogen infection (Zhou et al., 2022) and salicylic acid (SA) signaling, whereas *OsWKRY45*-mediated defense can be activated in the presence of SA signaling or pathogen infection reactions (Matsushita et al., 2013).

Known as a powerful gene editing tool, CRISPR/Cas9 has been widely used in rice to improve yield and quality traits, enhance disease resistance, and create male sterile rice lines to accelerate the process of hybrid rice breeding. For example, Yamauchi et al. used CRISPR/Cas9 technology to knock out the RBOHH gene and demonstrated its role in reducing Reactive oxygen species (ROS) accumulation in rice roots (Yamauchi et al., 2017); Li et al. used CRISPR/Cas9 to knock out four rice yield genes *Gn1a*, *DEP1*, *GS3*, and *IPA1* to assess their roles in rice yield (Li et al., 2016); Usman et al. (2020) improved the fragrance quality of rice by editing the *Badh2* gene (Usman et al., 2020); Zhou et al., (2022) edited the *Bsr-d1*, *Pi21*, and *ERF922* genes of LK638S and improved the resistance of LK638S to rice blast and bacterial blight (Zhou et al., 2022).

The evolution of *Magnaporthe oryzae* may lead to decreased or even completely lost rice blast resistance. Therefore, developing new rice lines with broad-spectrum resistance (BSR) to blast is necessary. However, bacterial leaf streak resistance is a quantitative trait controlled by multiple quantitative trait loci, and it is difficult to effectively select *Xoc*-resistant varieties by traditional breeding (Bossa-Castro et al., 2018; Xie et al., 2021). CRISPR/Cas9 gene editing technology has advantages in high efficiency, simple operation, affordable cost, and the ability to simultaneously edit multiple targets. CRISPR transgenic progeny can be screened for homozygous mutants without T-DNA insertion, reducing breeding time and labor costs significantly. In this study, CRISPR/Cas9 technology was used to edit the TALE-binding region of the susceptibility gene *OsSULTR3;6* and the second exon of *Pi21* gene in the high-quality indica maintainer line 58B, which simultaneously improved the resistance to rice blast and bacterial leaf streak. This work provides new and interesting breeding materials with both broad-spectrum blast resistance and bacterial leaf streak resistance.

## Materials and methods

### Material and pathogen materials

In this experiment, the indica maintainer line 58B preserved in Guangxi University was selected as the recipient material. This material has high quality and yield but is susceptible to rice blast and bacterial leaf streak. All experimental materials were independently planted in a planting pool in the rice net room or planting pond of Guangxi University. The CRISPR/Cas9 gene editing system used in this experiment was provided by YL, the South China Agricuture University. *Xoc* GX01 used was from in Guangxi University; *M. oryzae* H322 was a strain of rice blast isolated and preserved in the experimental field of Guangxi University by HP’s laboratory. The list of primers used in the study is shown in **Supplementary Table 1**.

### Vertor construct and rice transformation

Target sites of *Pi21* and *OsSULTR3;6*-EBE were selected by the CRISPR-Genome Editing (GE) (http://skl.scau.edu.cn/home). The target sites were introduced separately into the promoter and the Single guide RNA (sgRNA) using overlapping PCR. Subsequently, the promoter-target-sgRNA units were assembled into the CRISPR/Cas9 vector, following the method described by Zeng et al. (Zeng et al., 2018). The validated CRISPR/Cas9 plasmid was transformed into Agrobacterium tumefaciens EHA105, which was then used for rice transformation of the 58B variety (**Supplementary Figure 1**). Specific primer pairs Cas9-F/Cas9-R and HPT-F/HPT-R were used to confirm T_0_ transgenic-positive plants. *Pi21*-TF/*Pi21*-TR and *OsSULTR3;6*-TF/*OsSULTR3;6*-TR were used to amplify the genomic regions containing each target site, and the amplified products were followed for Sanger sequencing in T_0_ and T_1_ generations. The sequencing results were analyzed to determine the target mutation by an online tool DSDecodeM (http://skl.scau.edu.cn/dsdecode/) (Liu et al., 2015). Transgene-free plants were identified using the primer pairs Cas9-F/Cas9-R and HPT-F/HPT-R and determined by both showing negative amplification (**Supplementary Figure 3**). The genomic DNA was amplified with specific primers, and the amplified products were sequenced. The sequencing results were compared with the The Rice Annotation Project-Database (RAP-DB) sequence to analyze the off-target of each gene.

### *Magnaporthe oryzae* and Xoc inoculation

*Magnaporthe oryzae* H322 was used for inoculation in this experiment. The activated strain was transferred to oat medium for culture, placed in a 28°C incubator, and exposed to light for 24 h a day for 10 days to induce sporulation. Before inoculation, rice was selected with consistent growth to the three-leaf stage and the suspension prepared (the spore concentration was adjusted to 1 × 10^5^/mL, the total number of spores is about 30 in 16 middle squares of the blood count plate). The seedlings were sprayed evenly with the suspension, three biological replicates per strain. After inoculation, a layer of black film was placed on the transparent plastic film for shading and treatment for 36 h. After the treatment, the black film was removed, and it was kept moist for 5 days to investigate the incidence and lesions.

At the tillering stage, bacterial leaf streak inoculation was performed on transgene-free homozygous mutant. A single colony was selected from the streak-activated GX01 strain on NA medium (5 g/L tryptone, 1 g/L beeg extract, 1 g/L yeast extract, 10 g/L sucrose, 17 g/L agar, PH 6.8–7.0), and put into 500 mL of NB medium, which was cultured at 28°C at 180 rpm for 2 days. The OD_600_ value of bacterial suspension ranged between 0.6 and 0.8. GX01 was inoculated by acupuncture at the tillering stage (6 weeks) of rice (**Supplementary Figure 4**). The disease infection was investigated 14 days after inoculation and photographed.

### Gene expression difference analysis

Total RNA was extracted from fresh leaves using the FastPure Universal Plant Total RNA Isolation Kit (catalog no. RC411, Vazyme). The extracted RNA reverse-transcribed into Complementary DNA (cDNA), and Quantitative real-time (qRT)-PCR was performed. The gene expression levels of *pi21* and *Ossultr3;6* were detected in the mutant and wild types.

### Measurement of main agronomic traits

Wild-type (58B) and mutant lines were planted in Guangxi University. Each line was planted in four rows with eight plants in each row. At the maturity stage, five plants were randomly selected to investigate the plant height, effective panicle number, panicle length, number of grains per panicle, and 1,000-grain weight. Then, the data were analyzed using Excel and IBM SPSS Statistics 20.

## Results

### CRISPR/Cas9-mediated targeted mutagenesis of *Pi21* and *OsSULTR3;6*-EBE genes

**Figure 1:**
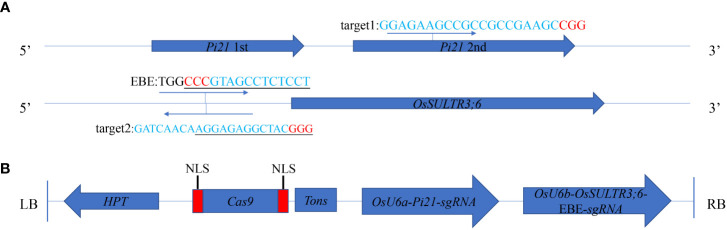
CRISPR/Cas9-mediated targeted mutagenesis of *Pi21* and *OsSULTR3;6*-EBE. **(A)** Target sites of CRISPR/Cas9. One target was chosen in the in the second exon of the *Pi21* gene; another target was chosen around effector-binding element (EBE) in the promoter region of *OsSULRT3;6* gene; the PAM sequences were marked in red. **(B)** The expression CRISPR/Cas9 vector. OsU6a and OsU6b, rice promoter; HPT, hygromycin; NLS, nuclear localization signal; Tons, the terminator; LB and RB, left border and right border, respectively.

To generate *Pi21* and *OsSULTR3;6*-EBE mutants, two sgRNAs that are in the second exon of the *Pi21* gene (*LOC_Os04g32850*) and EBE in the promoter region of *OsSULRT3;6* gene (*LOC_Os01g52130*) were designed (**Figure 1A**). Two sgRNAs were constructed into the CRISPR/Cas9 vertor (**Figure 1B**), and the vector plasmid was used to transform 58B rice by Agrobacterium tumefaciens EHA105. All 15 transgenic seedlings of 58b were positive plants with a transformation rate of 100% (**Supplementary Figure 2**). There was only one transgenic plant in target *Pi21* that did not have a detected target mutation, with a frequency of 6.67%. The frequencies of heterozygous and biallelic mutations were also the same at 26.67%, and the frequency of homozygous mutations was as high as 40%. Only one transgenic plant in the *OsSULTR3;6*-EBE target was not mutated. There were five heterozygous and five biallelic plants with a frequency of 33.33%, respectively. There were four plants with homozygous mutations with a frequency of 26.67% (**Table 1**). The mutation types of *Pi21* and *OsSULTR3;6*-EBE both include base insertions, substitutions, and deletions, including mainly base deletions (43.33% and 40%, respectively; **Table 2**).

**Table 1:** Analysis of T_0_ mutation types.

No. of plants	Target site	Proportion of mutation types(%)			
		Wild type	Heterozygous	Biallelic	Homozygous
15	*Pi21*	6.67 (1)	26.67 (4)	26.67 (4)	40 (6)
	*OsSULTR3;6*-EBE	6.67 (1)	33.33 (5)	33.33 (5)	26.67 (4)

**Table 2:** Frequency of T_0_ mutation types.

Target site	Frequency of mutation types (%)			
	WT	Insertion	Substitution	Deletion
*Pi21*	20	10	26.67	43.33
*OsSULTR3;6*-EBE	23.33	23.34	13.33	40

WT, wild type.

CRISPR/Cas9 gene editing technology inevitably presents off-target phenomena, which will interfere with experimental results. To avoid off-target events as much as possible, the potential off-target sites were predicted through the CRISPR-GE website (**Table 3**), and specific primers were designed for amplification and sequencing to analyze the off-target rate of each target. *Pi21* has three off-target sites located in the CDS region of the gene, namely, *Os03g0349200*, *Os01g0184800*, and *Os09g0380300*, and the highest off-target index reached 0.557. *Os03g0349200* presumably encodes a cyclin-dependent kinase C-2 protein involved in the cell cycle; *Os01g0184800* presumably encodes a photoconductive protein; and *Os09g0380300* presumably encodes a cytochrome P450 family protein. The two off-target sites of *OsSULTR3;6*-EBE were in the CDS region of *Os03g0209500* and *Os11g0568600*, respectively, and the other off-target sites were located in the non-coding region, with the highest off-target index of 0.405. The protein encoded by *Os03g0209500* belongs to the zinc finger family of proteins, whereas *Os11g0568600* encodes a protein containing a THUMP domain. The specific functions of these two proteins are unknown. Specific primers were designed for the abovementioned off-target sites in the CDS region of the gene, the DNA of the homozygous plants of the T_0_ generation was extracted for PCR amplification, and then the off-target situation was analyzed by sequencing. The results showed that no off-target events were detected in the two homozygous seedlings of *58b* (**Supplementary Figure 5**).

**Table 3:** Potential off-target analysis of the two target sites.

Target site	Off-target sequence	Off-score	Gene	Region
*Pi21*	GGAGAAGAAGCCGCCGAAGC CGG	0.557	*Os03g0349200*	CDS
	GGAGAAGAAGACGCCGAAGC CGG	0.418	Null	Intergenic
	GGGGAACCTGCCGCCGAAGC CGG	0.368	*Os01g0184800*	CDS
	GGAGAAGCCGCCGTCGCAGC CGG	0.129	*Os09g0380300*	CDS
*OsSULTR3;6*-EBE	TAGCAACAAAGAAAAGCTAC GGG	0.405	Null	Intergenic
	GATCAACAAGAAGAGACTGC TGG	0.375	*Os03g0209500*	CDS
	GAGCAACGGGGAGAGGCTAC GGG	0.244	Null	Intergenic
	AACTAAAAAGGAGAGGCTAC TGG	0.231	*Os11g0568600*	Intron
	CAACAACAAGGAGGAGCTAC GGG	0.224	Null	Intergenic
	CATTAAGGAGGGGAGGCTAC GGG	0.142	Null	Intergenic
	AACAAACAAGGAGAGGCCAC CGG	0.139	Null	Intergenic
	GGTCCACAAGAAGAGGCGAC GGG	0.133	Null	Intergenic

### Knockout of single *Pi21* enhanced resistance to rice blast

**Figure 2:**
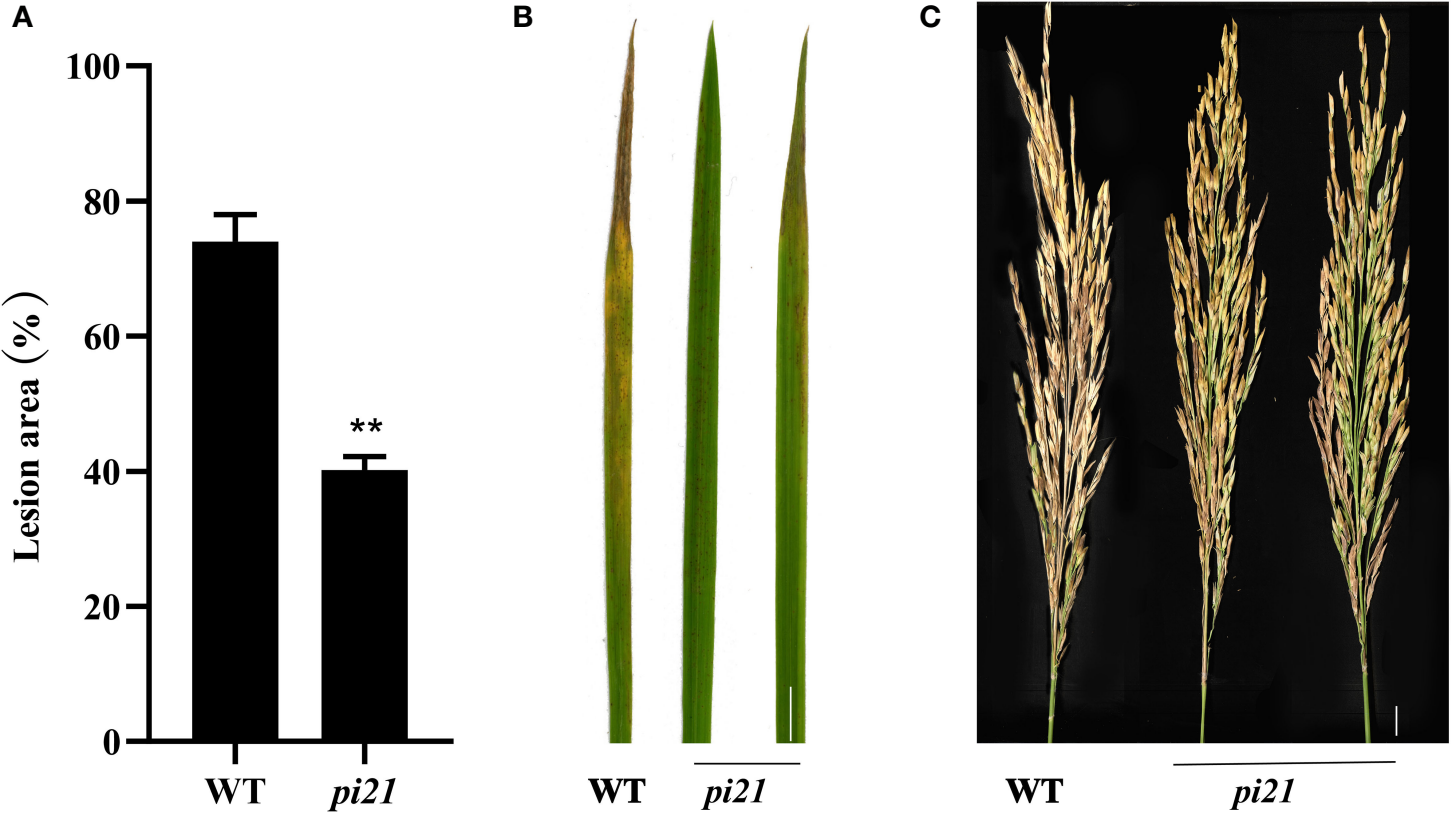
Enhanced blast resistance of the single *Pi21* mutant lines. **(A)** The percentage of lesion areas of rice blast (n = 3 leaves). **(B)** Rice mutant lines and wild-type 58B were tested for resistance to *M. oryzae* at the seedling stage. **(C)** The resistance to *M. oryzae* rice mutant lines and wild-type 58B was tested at rice reproductive stage. t-test: **P < 0.01.

The resistance to rice blast of a single *Pi21* homozygous mutant with a base insertion in the T_1_ generation was identified in seedling and maturity rice blast, three biological replications (**Table 4**). The single *Pi21* mutant was found to exhibit increased resistance to *M. oryzae* H322 at the seedling stage. To test whether enhanced blast resistance still existed at reproductive stage, the blast evaluation on rice panicles was conducted, and the mutants were transplanted in a field rice blast area in Wutang, Nanning. The results showed that percentage of diseased panicles in mutants were significantly lower than that in 58B (**Figure 2**).

**Table 4:** sgRNA sequence and mutations at the target site of *Pi21* in the T_1_ homozygous mutant.

Name	Edited gene sequence	Sanger chromotogram	Editing types
	*Pi21*		
58B	GGAGAAGCCGCCGCCGAAGC	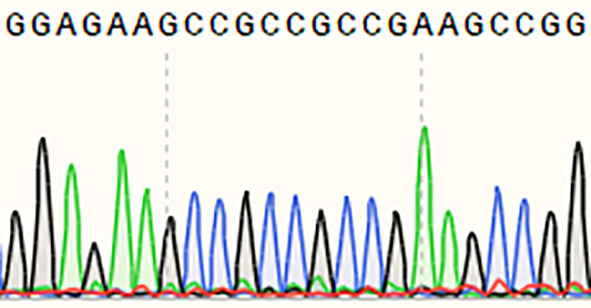	WT
*pi21*	GGAGAAGCCGCCGCCGATAGC	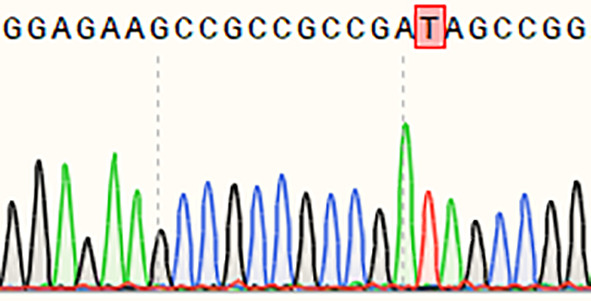	+1 bp

WT, wild type.

### Loss-of-function mutations of *OsSULTR3;6*-EBE increase resistance against *Xoc*

**Figure 3:**
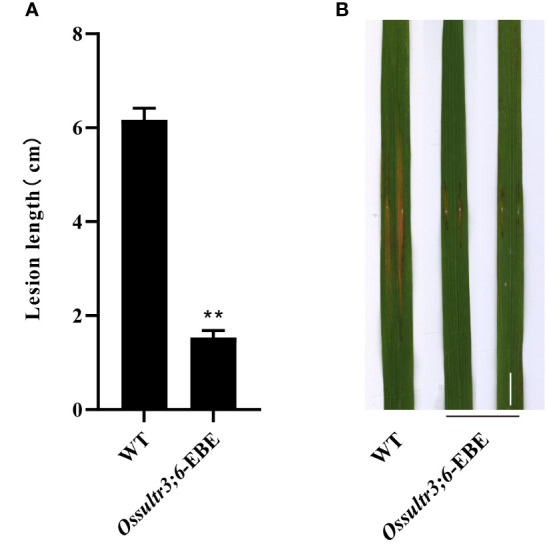
Enhanced bacterial leaf streak resistance of the single *OsSULTR3;6*-EBE mutant lines. **(A)** The percentage of lesion length of bacterial leaf streak (n = 3 leaves). **(B)** Rice mutant lines and wild-type 58B were tested for resistance to *Xoc* at the tillering stage. t-test: **P < 0.01.

A single homozygous *OsSULTR3;6*-EBE mutant line with a 4-base deletion was selected for further analysis (**Table 5**). The mutants were inoculated *Xoc* strain GX01 using the acupuncture method at the tillering stage, three biological replications. At 15 days after inoculation, disease length was about 75% shorter on the *Ossultr3;6-*EBE mutant than that on 58B (**Figure 3**). The results demonstrate that disrupting *OsSULTR3;6*-EBE significantly reduces susceptibility to *Xoc* in rice.

**Table 5:** Sequence at the target site of *OsSULTR3;6-*EBE in the T_1_ homozygous mutant.

Name	Edited gene sequence	Sanger chromotogram	Editing types
	*OsSULTR3;6-EBE*		
58B	GATCAACAAGGAGAGGCTAC	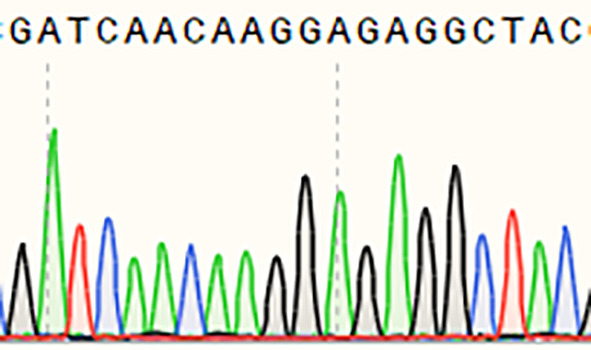	WT
*Ossultr3;6-*EBE	GATCAACAAGGAG****TAC	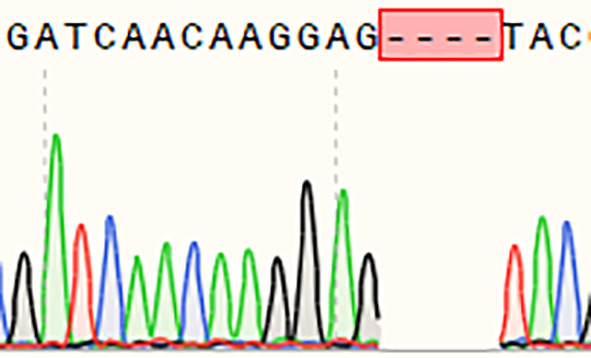	−4 bp

WT, wild type.

### The *Pi21/OsSULTR3;6*-EBE double mutant shows enhanced resistance to both *M. oryzae* and *Xoc*

**Figure 4:**
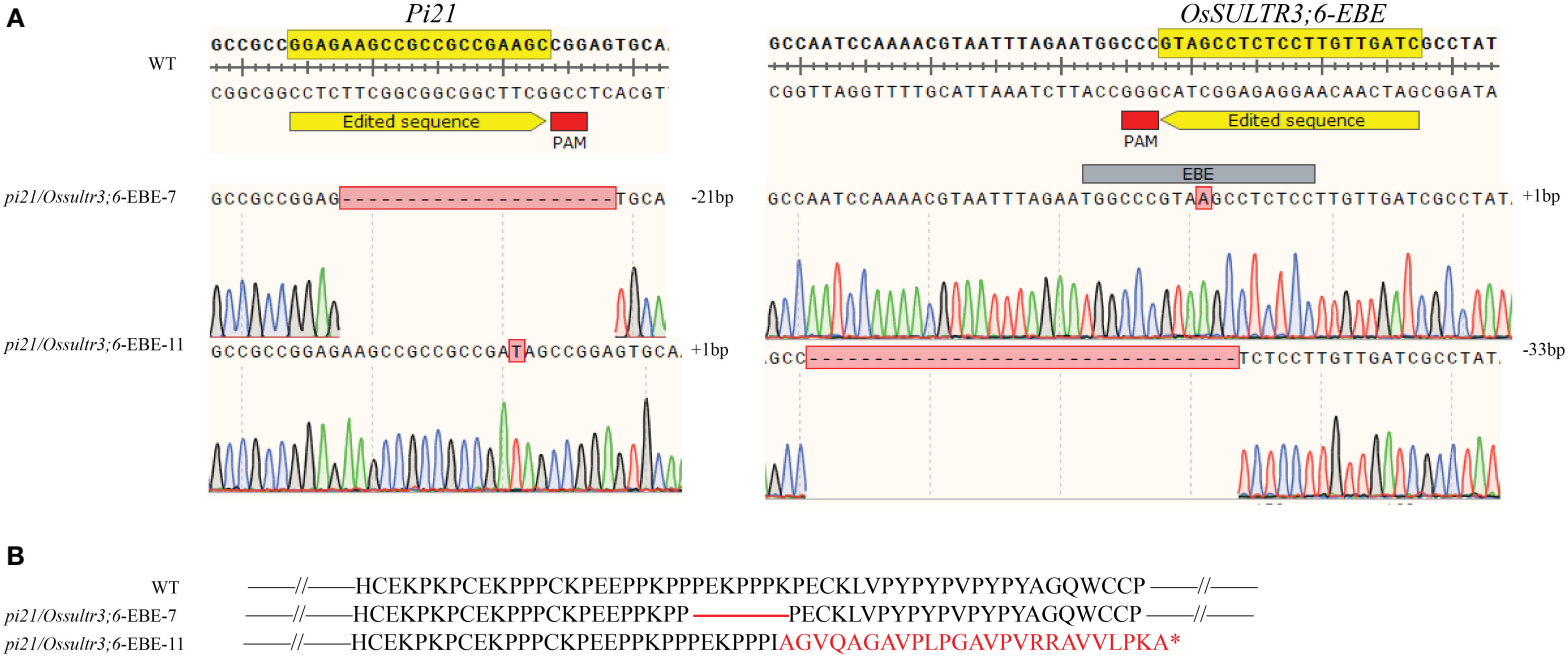
*pi21/Ossultr3;6*-EBE double mutation plants with different editing types **(A)** The mutated sequences of *Pi21* and *OsSULTR3;6*. The number of base deletion and insertion is shown by the mark of minus (−) and plus (+). **(B)** Amino acid variations of the Pi21 protein in the mutant. The red line indicates the missing protein. *indicates the termination of translation.

Two homozygous *pi21/Ossultr3;6*-EBE double mutant plants with different editing types in the T_1_ generation (*pi21*/*Ossultr3;6-*EBE-7 and *pi21*/*Ossultr3;6-*EBE-11) were used to identify the resistance (**Table 6**). *pi21*/*Ossultr3;6*-EBE-7 produced 21-bp deletion at *Pi21*, resulting in seven–amino acid “PEKPPPK” deletion in the Pi21 protein; 1-bp insertion at *OsSULTR3;6*-EBE did not cause a sequence change in the *OsSULTR3;6* gene and OsSULTR3;6 protein. *pi21*/*Ossultr3;6*-EBE-11 produced 1-bp insertion at *Pi21* and caused the frameshift in the *Pi21* coding region, generating the premature translation termination codon; *pi21*/*Ossultr3;6*-EBE-11 produced 33-bp deletion at *OsSULTR3;6*-EBE but did not directly affect the *OsSULTR3;6* gene and OsSULTR3;6 protein (**Figure 4**).

**Table 6:** sgRNA sequence and mutations at the target site of *Pi21/OsSULTR3;6*-EBE in the T_1_ homozygous mutant.

Name	Gene	Edited gene sequence	Editing types
58B	*Pi21*	GGAGAAGCCGCCGCCGAAGC	WT
	*OsSULTR3;6*-EBE	GATCAACAAGGAGAGGCTAC	WT
*pi21*/*Ossultr3;6-*EBE-7	*Pi21*	GGAG********************* TGC	−21 bp
	*OsSULTR3;6*-EBE	GATCAACAAGGAGAGGCTTAC	+1 bp
*pi21*/*Ossultr3;6*-EBE-11	*Pi21*	GGAGAAGCCGCCGCCGAAAGC	+1 bp
	*OsSULTR3;6*-EBE	GATCAACAAGGAGAGGCGTAC	+1 bp

WT, wild type.

**Figure 5:**
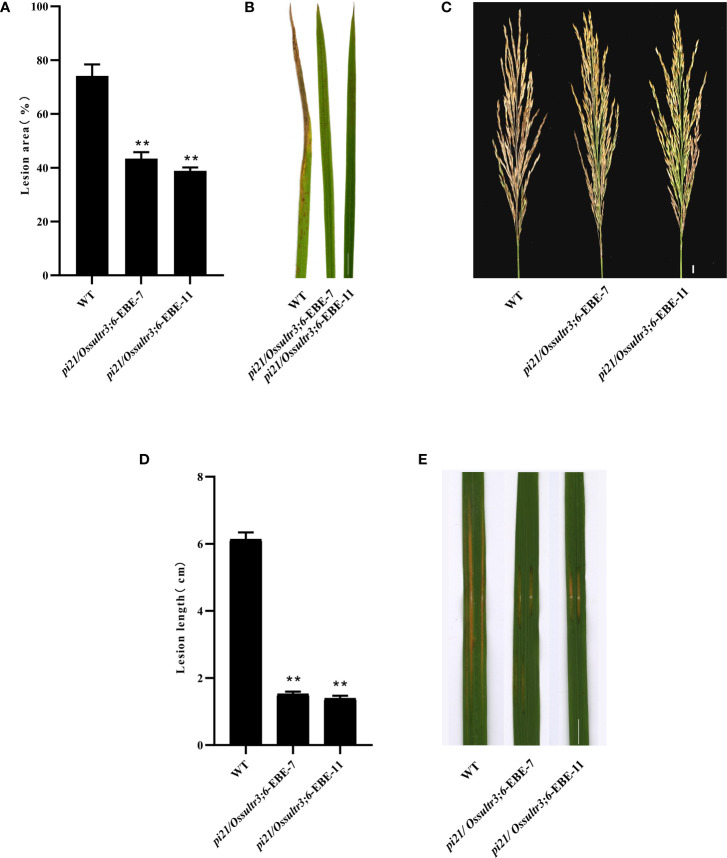
Bacterial leaf streak and rice blast resistance were enhanced in mutants. **(A)** The percentage of lesion areas of rice blast. **(B)** Rice mutant lines and wild-type 58B were tested for resistance to *M. oryzae* at the seedling stage. **(C)** Rice mutant lines and wild-type 58B were tested for resistance to *M. oryzae* at the mature stage. **(D)** The percentage of lesion length of bacterial leaf streak. **(E)** Rice mutant lines and wild-type 58B were tested for resistance to *Xoc* at the tillering stage. t-test: **P < 0.01.

To identify the blast resistance of the *pi21* in homozygous mutant, *pi21*/*Ossultr3;6*-EBE-7 and *pi21*/*Ossultr3;6*-EBE-11 were collected and inoculated *M. oryzae* H322 at the three-leaf stage by spraying. After 7 days, the area of lesions was counted, and the resistance to rice blast at the seedling stage was identified. Simultaneously, *pi21*/*Ossultr3;6*-EBE-7 and *pi21*/*Ossultr3;6*-EBE-11 were also planted in Wutang, Nanning, where the rice blast naturally occurred, and the rice blast resistance was identified after the rice was mature. The results showed that, compared with the wild type, the *pi21*/*Ossultr3;6*-EBE homozygous mutant had significantly decreased lesion area, indicating that knockout of the *pi21* gene significantly improved the rice blast resistance at seedling and mature stages (**Figures 5A–C**). To identify bacterial leaf streak resistance of homozygous mutants of *pi21*/*Ossultr3;6*-EBE, acupuncture was used to inoculate *Xoc* GX01, and the lesion length was measured and analyzed 15 days after inoculation. The results showed that, compared with the wild type, the length of the lesions of the *pi21*/*Ossultr3;6*-EBE-7 and *pi21*/*Ossultr3;6*-EBE-11 lines was significantly reduced, indicating that the knockout of the *OsSULTR3;6*-EBE had significantly improved resistance (**Figures 5D, E**).

### Expression analysis of *pi21*, *Ossultr3;6* genes and defense responsive genes

**Figure 6:**
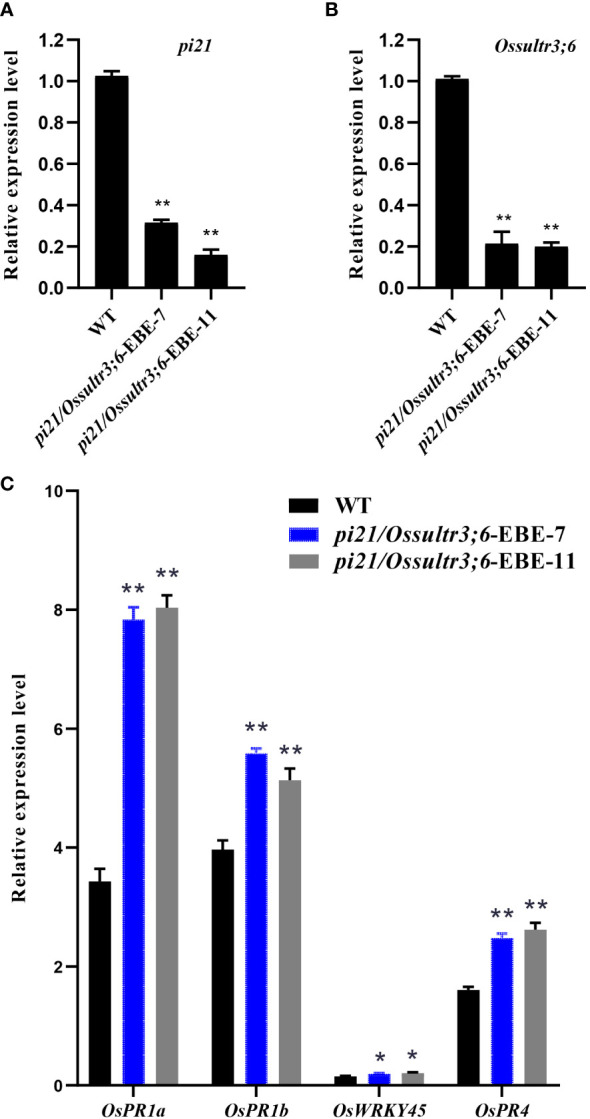
Expression analysis of *pi21*, *Ossultr3;6* genes and defense responsive genes. **(A)** Relative expression of *pi21*. **(B)** Relative expression of *ossultr3;6*. **(C)** Relative expression level of defense responsive genes after inoculation of rice blast. t-test: *P < 0.05 and **P < 0.01.

As susceptibility genes, the *Pi21* and *OsSULTR3;6* genes play an important role in the invasion of pathogens and promote rice disease through transcription and translation into corresponding proteins, and their expression levels have a huge impact on the degree of disease. To further study the effect of *pi21* and *Ossultr3;6* genes mutations on rice plant susceptibility, the expression levels of *pi21* and *Ossultr3;6* in the knockout mutants were detected. The wild type was used as the control, and *OsActin* was used as the internal reference gene for qRT-PCR detection. The results showed that the expression of the *pi21* gene of *pi21*/*Ossultr3;6*-EBE-7 and *pi21*/*Ossultr3;6*-EBE-11 plants was significantly decreased by compared with that of the wild type (**Figure 6A**). The *Ossultr3;6* gene expression levels of *pi21*/*Ossultr3;6*-EBE-7 and *pi21*/*Ossultr3;6*-EBE-11 were decreased, respectively, compared with that of the wild type (**Figure 6B**). The result showed that the *pi21* gene can significantly reduce its expression and improve the resistance to rice blast; after editing EBE, it can prevent the TALE secreted by *Xoc* from binding with EBE, thereby significantly reducing the expression of *Ossultr3;6* gene and achieving disease resistance.

After the pathogenic bacteria infect rice, the plant will initiate an immune response through the hormone signaling pathway. To study the role of the *pi21* in the pathogenic infection of rice, the leaves of the inoculated site were taken 24 h after inoculation, and the extracted RNA was used to detect the expression of SA signaling pathway marker genes *OsPR1a*, *OsPR1b*, *OsWRKY45*, and JA signaling pathway marker gene *OsPR4*. The results showed that the gene expression levels of the SA signaling pathway and the JA signaling pathway were significantly increased after rice blast inoculation (**Figure 6C**), which may be the reason why *pi21* showed disease resistance.

### Main agronomic traits of wild-type and mutant lines

**Figure 7:**
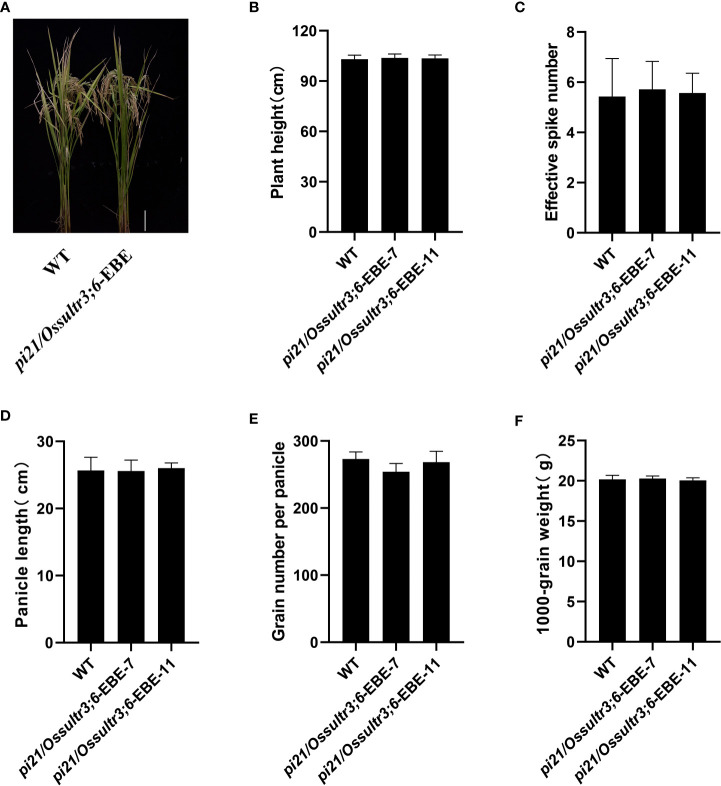
The agronomic traits of the mutants and the wild type (WT). **(A)** Phenotypes of *pi21*/*Ossultr3;6*-EBE mutant lines. **(B)** Plant height. **(C)** Effective spike number. **(D)** Panicle length. **(E)** Grain number per panicle. **(F)** 1,000-grain weight.

To study the effects of *Pi21* and *OsSULTR3;6* gene editing on the main agronomic traits of rice, the homozygous mutants without foreign genes in the T_2_ generation were chosen and planted in separate planting ponds. Their main agronomic traits were then statistically analyzed under the same growing conditions. *pi21*/*Ossultr3;6*-EBE-7 and *pi21*/*Ossultr3;6*-EBE-11 lines with both *Pi21* and *OsSULTR3;6*-EBE mutations did not differ significantly from the wild type in plant height, 1,000-grain weight, panicle length, number of grains per panicle, or effective panicle number, This results indicated that the *Pi21* and *OsSULTR3;6*-EBE mutation did not affect the main agronomic traits (**Figure 7**).

## Discussion

Currently, rice breeding should consider not only high yield but also high rice quality. Rice blast and bacterial leaf streak are the main diseases that seriously harm the yield and quality of rice (Liu et al., 2014; Asibi et al., 2019). With the rapid development of CRISPR/Cas9-mediated genomic technologies, modifying S genes to produce new varieties with BSR has become feasible for many crops (Xu et al., 2019). For example, the CRISPR/Cas9 knockout mutant of the rice S gene *Pi21* is resistant to *M. oryzae* (Nawaz et al., 2020). 58B is an Indica rice variety independently bred by our laboratory for many years. 58B originated from yexiang B/IR58025B//43B/DP15; among them, yexiang B and 43B are indica rice maintainer line from China. IR58025B is derived from the International Rice Research Institute indica rice maintainer line. DP15 is a common wild rice (*O. rufipogon Griff*). It has outstanding advantages, high rice quality, first grade rice, and good taste. It has short stalks, strong tillering power, narrow leaves, upright, good plant shape, strong compatibility, strong hybrid advantage, and good productivity. However, it is susceptible to rice blast and bacterial leaf streak, which affects yield and quality.

To address this issue, in the study, CRISPR/Cas9 technology was used to precisely edit both the BSR rice blast gene *Pi21* and the promoter region EBE of the susceptibility gene *OsSULRT3;6*, which produced to the single *pi21* mutants, single *Ossultr3;6*-EBE mutants, and *pi21/Ossultr3;6*-EBE double mutants. After inoculation of *M. oryzae* and *Xoc*, the results showed that single mutants of the S gene *Pi21* or *OsSULTR3;6*-EBE can enhance resistance to *M. oryzae* or *Xoc*, and this is consistent with the previous results (Nawaz et al., 2020; Xu et al., 2020). Noteworthy, when both *Pi21* and *OsSULTR3;6*-EBE were edited, the *pi21/Ossultr3;6*-EBE double mutant has resistance against both *M. oryzae* and *Xoc*. Similar to the resistance conferred by the single mutants, no superimposed or attenuated effects were found. The mutant that was planted in the rice blast–infected field also demonstrated heightened resistance against rice blast. However, the current shortage of *M. oryzae* or *Xoc* strains in the laboratory hinders the ability to determine whether the *pi21/Ossultr3;6*-EBE double mutants exhibit BSR. This crucial investigation will need to be conducted in the future.

Disruption of the S gene can cause other effects including reduced growth, low fertility, and reduced tolerance to other stresses (Zaidi et al., 2018). For example, knockdown of the S gene *OsSWEET11* in rice significantly reduced the sucrose concentration in the embryo sac of the mutants, resulting in seed germination deficiency (Ma et al., 2017). The *Xa13* gene controls not only disease resistance but also reproductive growth in rice. If its expression is suppressed while enhancing resistance to strain PX099A, then it can also cause pollen abortion in rice and reduce the fruiting rate (Yang et al., 2018). To eliminate this side effect, Li used CRISPR/Cas9 to edit the *Xa13* promoter to obtain *Xoo* resistant rice with normal fertility (Li et al., 2011). *Xoc* introduces TALE into the plant cell through the type III secretion system, which recognizes and binds EBEs in the promoter region of the host susceptibility gene, activating the transcriptional expression of the susceptibility gene and making the host susceptible to disease (Hui et al., 2019). At present, there are still few studies on the genes corresponding to TALE in *Xoc*. The sulfate transporter protein gene *OsSULTR3;6* is a susceptible gene, which can be bound by Tal2g, one TALE of *Xoc*, and cause bacterial leaf streak in host rice (Cernadas et al., 2014). By modifying the Tal2g-binding region (EBE) of the *OsSULTR3;6* gene, we were able to obtain homozygous mutants that had a 1-bp base insertion and a 33-bp base deletion within the EBE region. It is important to note that the actual sequence of the *OsSULTR3;6* gene itself remained unchanged. The bacterial leaf streak resistance was identified by acupuncture method, and both mutant strains showed significantly lower spot length and higher resistance than wild-type 58B, indicating that editing the EBE region of the susceptibility gene could effectively improve the resistance of rice to bacterial leaf streak, and this is consistent with the previous results (Xu et al., 2020). In the study, higher levels of SA signaling related genes *OsPRla*, *OsPRlb*, *OsWRKY45* and JA signaling related gene *OsPR4* were detected in mutants than in wild-type plants when the plants were infected with *M. oryzae* (**Figure 6**). These results suggest that the enhanced resistance of the mutant to rice blast may be associated with the activation of SA and JA signaling transduction genes. In the study, five key agronomic traits were evaluated in the field for the *pi21/Ossutr3;6*-EBE double mutants and observed no significant differences in these traits between the mutant and WT plants.

In conclusion, our work provides a rapid and effective approach to breed rice varieties resistant to rice blast and bacterial leaf streak, which could significantly accelerate the breeding of rice varieties with multiple disease resistance.

## Data availability statement

The original contributions presented in the study are included in the article/**Supplementary Material**. Further inquiries can be directed to the corresponding authors.

## Author contributions

RL, SC, and FL designed and supervised the research. JY, YF, and HW performed most experiments. JY, YF, and XG analyzed date. JY, YF, NZ, EM, and RL wrote the paper. HP, SH, YH, BQ, YL, SC, and RL provided resources. All authors contributed to the article and approved the submitted version.
