# Supplementary material for: Improvement of resistance to rice blast and bacterial leaf streak by CRISPR/Cas9-mediated mutagenesis of Pi21 and OsSULTR3;6 in rice (Oryza sativa L.)
Source: Front Plant Sci. 2023 Jul 17;14:1209384. doi: 10.3389/fpls.2023.1209384 (PMC10389665; doi:10.3389/fpls.2023.1209384)
Supplement: Supplementary file 1 [file DataSheet_1.docx]

Supplementary Material

Improvement of Resistance to Rice Blast and Bacterial Leaf Streak by CRISPR/Cas9-mediated Mutagenesis of *Pi21* and *OsSULTR3;6* in Rice (*Oryza Sativa* L.)

**Jinlian Yang^1^**^†^**, Yaoyu Fang^1^**^†^**, Hu Wu^1^, Neng Zhao^1^, Xinying Guo^1^, Enerand Mackon^1^, Haowen Peng^1^, Sheng Huang^2^, Yongqiang He^1^, Baoxiang Qin^1^, Yaoguang Liu^3^, Fang Liu^1^,** Shengwu Chen^4*^, **Rongbai Li^1*^**

*** Co-correspondence:** Rongbai Li: lirongbai@126.com, Shengwu Chen: 692792063@qq.com

# Supplementary Table1 List of primers in this study.

| Primer name | Primer sequence (5'-3') | Purpose |
| --- | --- | --- |
| *Pi21-*gRT1 | GAGAAGCCGCCGCCGAAGCGTTTTAGAGCTAGAAAT | Vector construction |
| *Pi21*-OsU6a-T1 | GCTTCGGCGGCGGCTTCTCCGGCAGCCAAGCCAGCA |  |
| *OsSULTR3;6*-gRT2 | ATCAACAAGGAGAGGCTACGTTTTAGAGCTAGAAAT |  |
| *OsSULTR3;6*-OsU6bT2 | GTAGCCTCTCCTTGTTGATCAACACAAGCGGCAGC |  |
| U-F | CTCCGTTTTACCTGTGGAATCG |  |
| gR-R | CGGAGGAAAATTCCATCCAC |  |
| Pps-R | TTCAGAGGTCTCTACCGACTAGTCACGCGTATGGAATCGGCAGCAAA |  |
| Pgs-2 | AGCGTGGGTCTCGTCAGGGTCCATCCACTCCAAGCTC |  |
| Pps-2 | TTCAGAGGTCTCTCTGACACTGGAATCGGCAGCAAAGG |  |
| Pgs-L | AGCGTGGGTCTCGCTCGACGCGTATCCATCCACTCCAAGC |  |
| SP-L1 | GCGGTGTCATCTATGTTACTAG |  |
| SP-R | TGCAATAACTTCGTATAGGCT |  |
| Cas9-F | CTGACGCTAACCTCGACAAG | Positive plants detection |
| Cas9-R  HPT-F  HPT-R | CCGATCTAGTAACATAGATGACACC  AAATCCGCGTGCACGAGGT  TCGTTATGTTTATCGGCACTTTGCA |  |
| *pi21*-TF | CGAGGCAAGGAAAGGAGCAG | Target detection |
| *pi21*-TR | AAGTTCGACCCGGAGAAGCT |  |
| *osSultr3;6*-TF | CAAGGAGATCGAACGGTGCC | Target detection |
| *osSultr3;6*-TR | TCGAGTCGAGAGCGTTTTGC |  |
| *osActin*-qF | GAGTATGATGAGTCGGGTCCAG | qtPCR reference gene |
| *osActin*-qR | ACACCAACAATCCCAAACAGAG |  |
| *pi21*-qF | TCCTTGCCTCGGAAATCGG | qtPCR |
| *pi21*-qR | TATACAGCCCACACAGCACAC |  |
| *osSultr3;6*-qF | CGCCATTCATGGTTGTGTC |  |
| *sSultr3;6*-qR | CGTCTTCATGTGCCTGGACT |  |


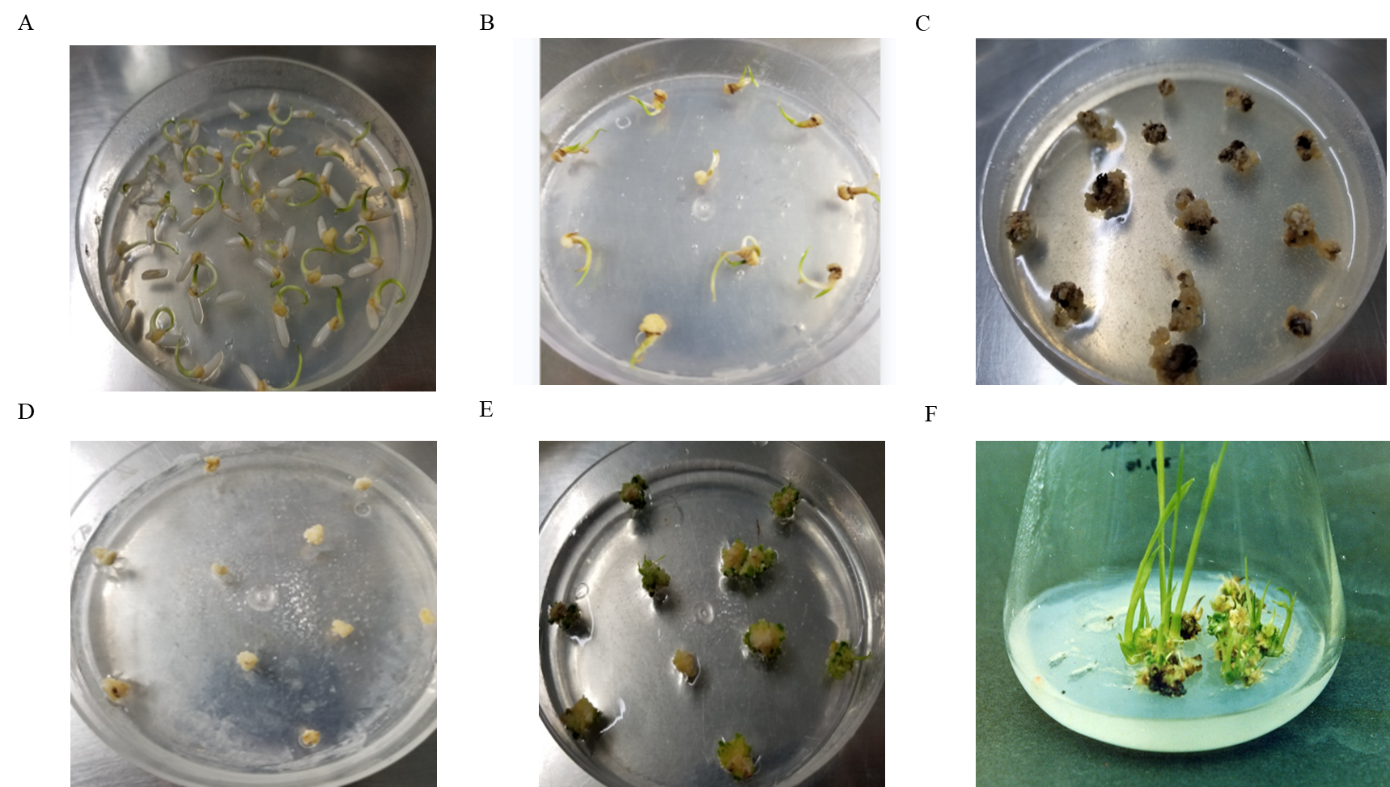


**Supplementary Figure 1.** Genetic transformation of rice materials. (A) Callus induction. (B) Callus screening. (C) The first screening of callus. (D) The second screening of callus. (E) Stage of inducing callus differentiation. (F) Plantlet differentiation and rooting.


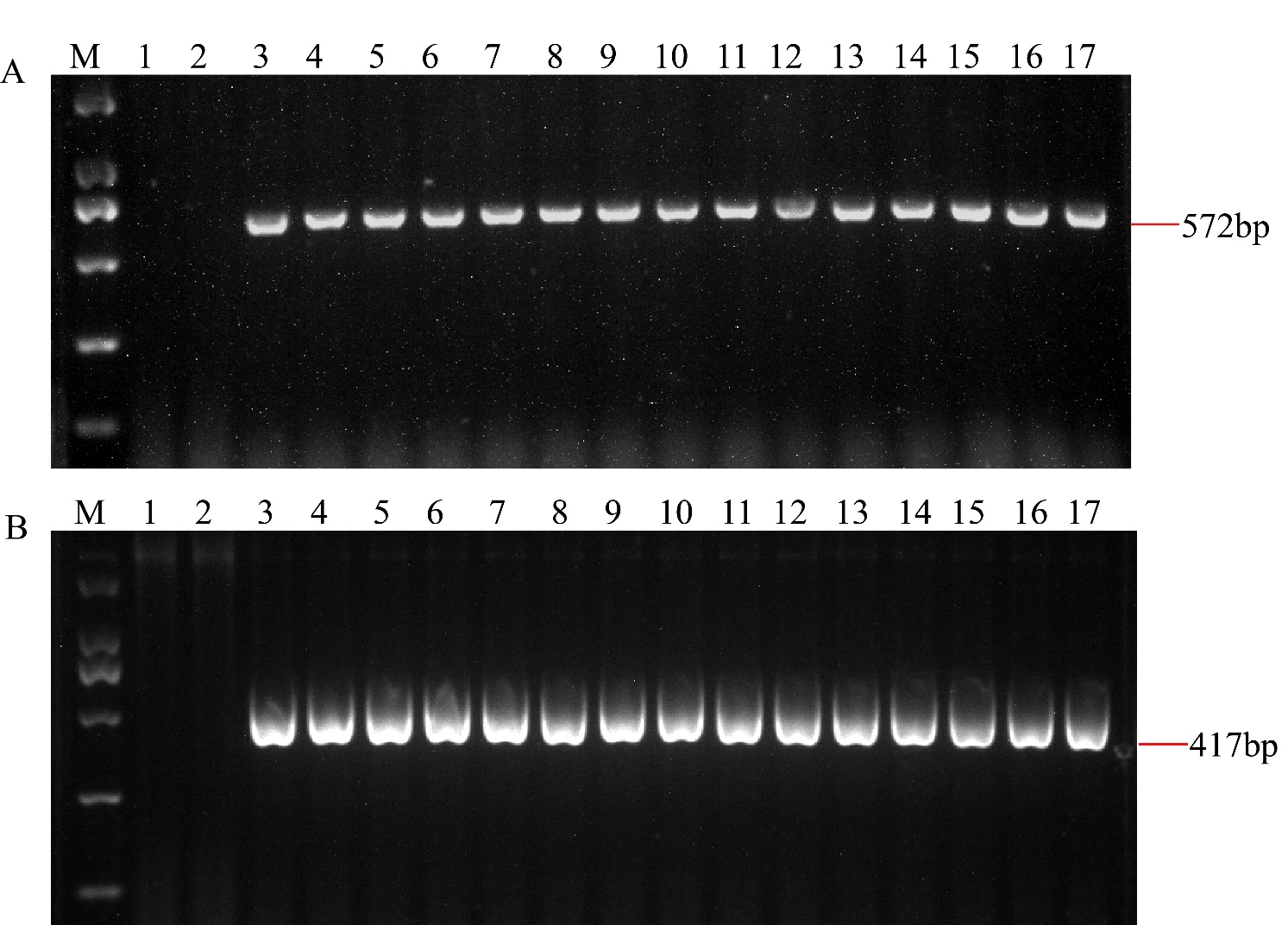
**Supplementary Figure 2.** PCR-based identification of T_0_ transgenic plants. M: DL 2000 DNA Marker; 1: ddH_2_O; 2: Wild type; 3-17: T_0_ generation transgenic plants. A: Primer Cas9-F/Cas9-R; B: Primer HPT-F/HPT-R.


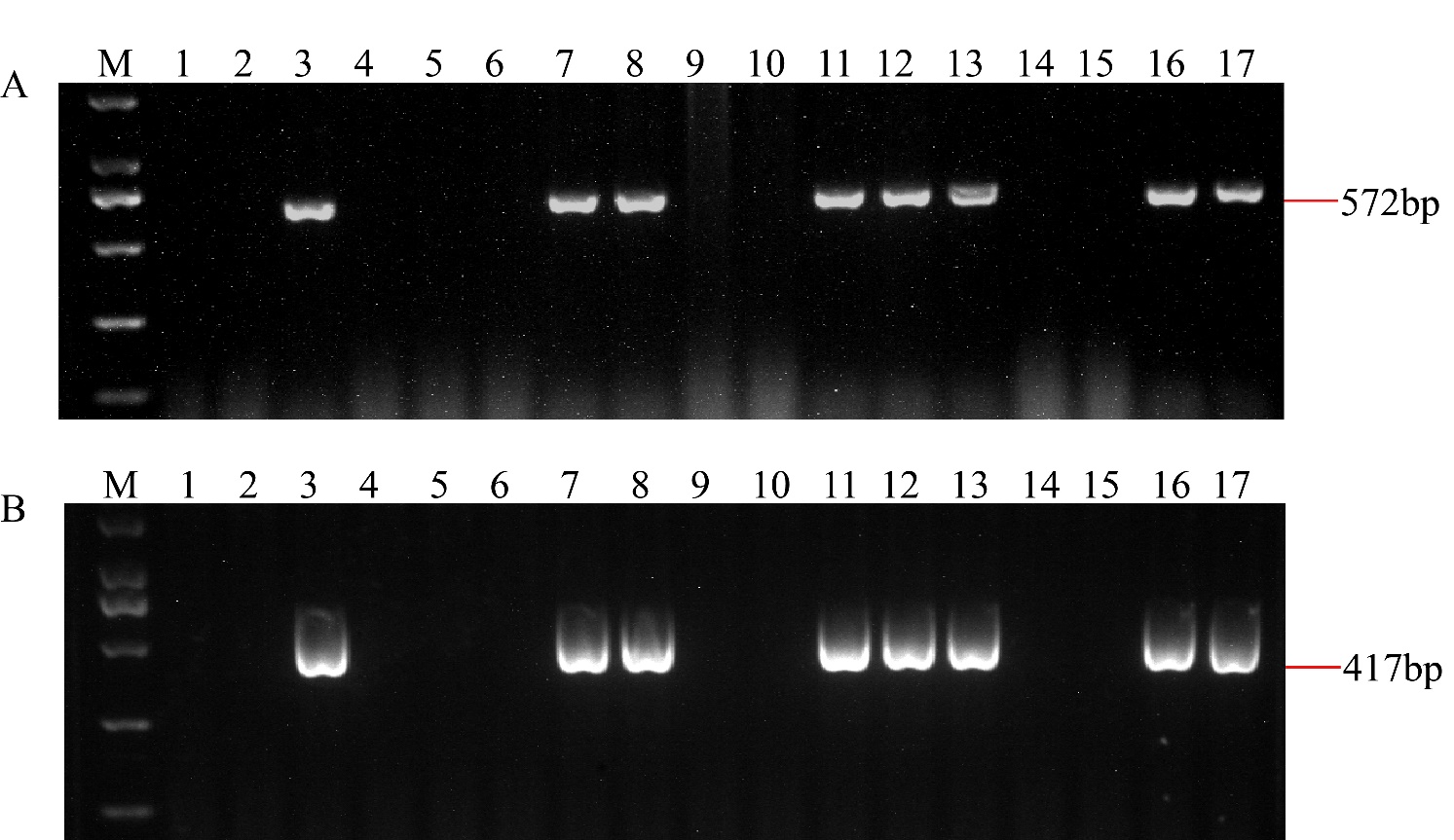


**Supplementary Figure 3.** Identification of transgene-free plants. M: DL 2000 DNA Marker; 1: ddH_2_O; 2: Wild type; 3: Positive control; 4-17: T_1_ generation transgenic plants. A: Primer Cas9-F/Cas9-R; B: Primer HPT-F/HPT-R.

**
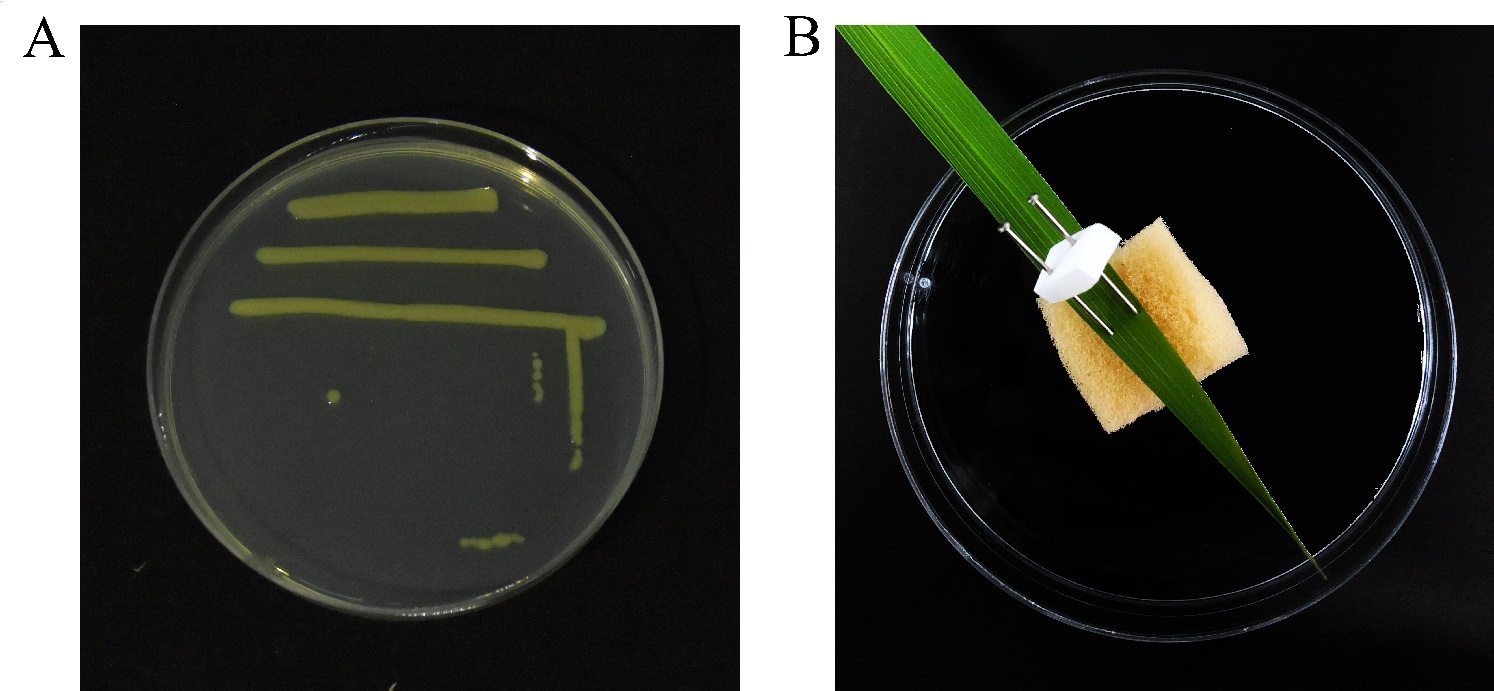
**

**Supplementary Figure 4.** Determination of bacterial leaf streak. (A) *Xoc*, GX01. (B) Inoculation method, acupuncture.


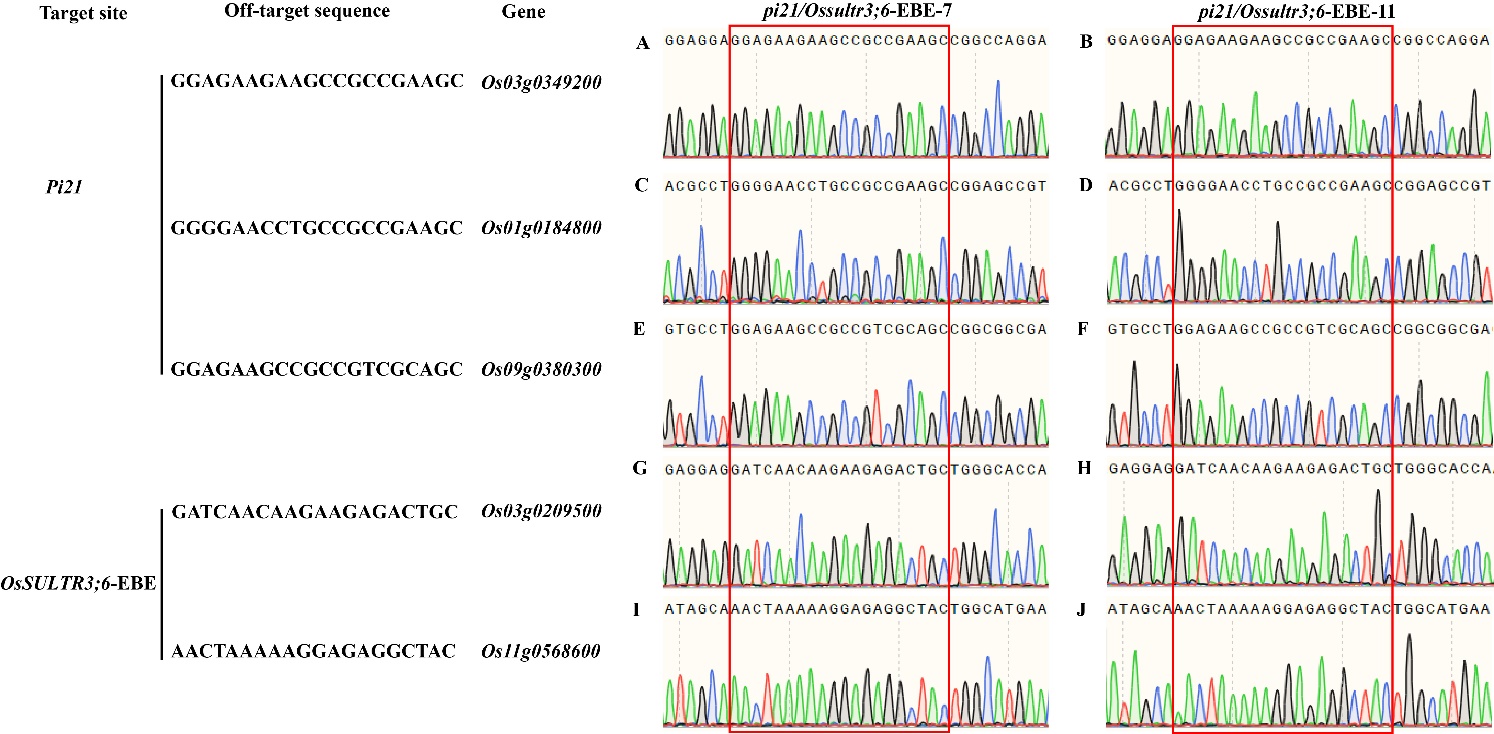


**Supplementary Figure 5.** Off-target detection of *Pi21* and *OsSULTR3;6*-EBE. No off-target events were detected in the two homozygous mutant plants, *pi21*/*Ossultr3;6*-EBE-7 and *pi21*/*Ossultr3;6*-EBE-11. The red box area is off-target sequence. (A) and (B) Sequencing results of *Os03g0349200*. (C) and (D) Sequencing results of *Os01g0184800*. (E) and (F) Sequencing results of *Os09g0380300*. (G) and (H) Sequencing results of *Os03g0209500*. (I) and (J) Sequencing results of *Os11g0568600*.
